# The impact of an “acute dialysis start” on the mortality attributed to the use of central venous catheters: a retrospective cohort study

**DOI:** 10.1186/1471-2369-13-72

**Published:** 2012-07-30

**Authors:** Karthik K Tennankore, Steven D Soroka, Bryce A Kiberd

**Affiliations:** 1 Department of Medicine, Division of Nephrology, University of Toronto, University Health Network/Toronto General Hospital, 21 Carlton Street, Unit 1405, Toronto, ON, Canada; 2 Division of Nephrology, Department of Medicine, Dalhousie University, Halifax, NS, Canada

**Keywords:** Vascular, Mortality, Dialysis, Catheter, Incident, Access, Fistula, Peritoneal, Acute, Chronic

## Abstract

**Background:**

Central venous catheters (CVCs) are associated with early mortality in dialysis patients. However, some patients progress to end stage renal disease after an acute illness, prior to reaching an estimated glomerular filtration rate (eGFR) at which one would expect to establish alternative access (fistula/peritoneal dialysis catheter). The purpose of this study was to determine if exclusion of this “acute start” patient group alters the association between CVCs and mortality.

**Methods:**

We conducted a retrospective cohort study of 406 incident dialysis patients from 1 Jan 2006 to 31 Dec 2009. Patients were classified as acute starts if 1) the eGFR was >25 ml/min/1.73 m^2^, ≤3 months prior to dialysis initiation and declined after an acute event (n = 45), or 2) in those without prior eGFR measurements, there was no supporting evidence of chronic kidney disease on history or imaging (n = 12). Remaining patients were classified as chronic start (n = 349).

**Results:**

98 % and 52 % of acute and chronic starts initiated dialysis with a CVC. There were 148 deaths. The adjusted mortality hazard ratio (HR) for acute vs. chronic start patients was 1.84, (95 % CI [1.19-2.85]). The adjusted mortality HR for patients dialyzing with a CVC compared to alternative access was 1.19 (95 % CI [0.80-1.77]). After excluding acute start patients, the adjusted HR fell to 1.03 (95 % CI [0.67-1.57]).

**Conclusions:**

A significant proportion of early dialysis mortality occurs after an acute start. Exclusion of this population attenuates the mortality risk associated with CVCs.

## Background

Incident use of central venous catheters (CVCs) as initial hemodialysis (HD) access is associated with increased mortality in prospective studies and large registry analyses [1-4]. While CVCs are more common in late referrals [5], they are associated with a higher mortality risk compared to arteriovenous fistulas (AVFs) even after adjusting for timing of referral [1]. Dialysis with a CVC is also associated with a higher risk of death compared to peritoneal dialysis (PD) [6]. As a result of the association between CVCs and mortality in HD, guidelines recommend the use of an AVF as initial HD access [7-9]. Recognizing that AVFs require time to mature, guidelines also suggests that AVF creation should occur in a timely fashion. Timely initiation of PD in patients with chronic kidney disease (CKD) is also emphasized [10]. Despite these recommendations, most incident patients continue to dialyze with a CVC [2,6].

With a few exceptions [11,12], CVCs are used as initial dialysis access in patients with acute renal failure/acute kidney injury (AKI) or acute on early stage chronic kidney disease (ACKD), as timely placement of alternative access is not a consideration in these patients. As high as 16 % and 28 % of patients do not recover kidney function after acute renal failure [13] and ACKD [14], respectively. In addition, progression to end stage renal disease (ESRD) in both these situations is itself associated with increased mortality [13,14].

A previous cohort study identified that an “emergency dialysis start” confounded the association between CVCs and mortality [15]. However, this study did not separately analyze patients with an acute decline in GFR from patients presenting emergently with late stage CKD. A recent case series identified that a rapid decline in estimated glomerular filtration rate (eGFR; in patients with an eGFR >30 ml/min/1.73 m^2^) may be an unavoidable cause of incident use of CVCs, but did not examine its association with mortality [16].

Therefore, in a retrospective cohort of incident dialysis patients, the purposes of this study were to explore the following:

1) Determine the proportion of incident dialysis patients who develop ESRD after a permanent loss of GFR in the context of an acute illness event (“acute start”).

2) Identify if patients who start dialysis under this circumstance are at an increased risk of mortality.

3) Determine if CVCs are associated with mortality, and if this association persists after excluding patients with an illness induced permanent GFR loss.

We hypothesize that CVC mortality may be overestimated without considering the influence of an acute start.

## Methods

### Population

We conducted a retrospective cohort study consisting of 481 consecutive, adult (> 18 years) ESRD dialysis starts at a tertiary care center in Halifax, Nova Scotia, Canada over a period of 48 months (1 Jan 2006 to 31 Dec 2009). We chose this time frame to coincide with the availability of electronic patient/ESRD database records at our institution (which commenced just prior to Jan 2006), and ensure a minimum of one year of follow-up from the last date of study entry. Patients were excluded if they transferred to another regional dialysis unit (Prince Edward Island, Cape Breton or Yarmouth) or out of province after having received only their initial HD in Halifax.

### Exposure assessment

Patients were classified as “acute start” under the following circumstances:

1) eGFR by MDRD >25 ml/min/1.73 m^2^ ≤ three months prior to dialysis initiation that declined in the context of an acute illness event requiring hospitalization.

2) Acute illness event leading to dialysis initiation in patients without prior eGFR measurements and no suggestion of chronic kidney disease on history or renal ultrasound [16].

Remaining patients were classified as “chronic start”:

1) eGFR by MDRD ≤25 ml/min/1.73 m^2^ > three months prior to dialysis initiation.

2) eGFR by MDRD >25 ml/min/1.73 m^2^ ≤ three months prior to dialysis initiation without a documented acute illness event.

3) Late presentation of ESRD, suggested by ultrasound evidence of bilateral small kidneys in individuals without prior eGFR measurements [17].

The definition of an acute start was made on the assumption that most patients are not referred for evaluation and/or placement of AVF or PD catheter access prior to reaching an eGFR of <25 ml/min/1.73 m^2^. This is consistent with previously published guideline recommendations on appropriate referral for permanent access placement [7,9]. Therefore, we expected that the vast majority of patients with an eGFR of >25 ml/min/1.73 m^2^ who progressed to ESRD within three months would be unexpected dialysis starts without established or planned alternative access.

Access at dialysis initiation was classified as a central venous catheter (CVC), peritoneal dialysis (PD) catheter or arteriovenous fistula (AVF). There was only one arteriovenous graft at this center and for the purpose of this study it was classified as a fistula. AVF access required completion of one dialysis treatment with the AVF. PD access required successful completion of the training program. No incident patients were on combination therapy. All patient definitions were independently assessed by a nephrologist and nephrology fellow, subsequent to which consensus was achieved.

### Baseline data

Clinical data, including cause and date of death was accessed from individual patient Canadian Organ Replacement Registry (CORR) forms and supplemented with electronic patient record data. In addition to cause of ESRD, date of dialysis initiation and access type, a Charlson Comorbidity Index (CCI) was calculated at the time of dialysis start in all incident patients. This index allocates different point scores to 19 individual medical conditions depending on the risk of mortality associated with each condition [18]. We also collected several laboratory parameters including creatinine, phosphate, hemoglobin and albumin in all patients at baseline. All assays were performed by the institution’s laboratory. We manually calculated a four variable modified diet in renal disease (MDRD) eGFR at dialysis initiation in all patients, using available creatinine measurements. In addition, we used electronic chart and laboratory systems review to identify available outpatient creatinine values (from which we calculated eGFRs) at 1, 3, 6, 9 and 12 months prior to dialysis initiation. We allowed a ± two-week time frame at each time interval. Finally, the acute event prompting dialysis initiation in acute start patients was determined using electronic patient records.

### Outcome

We initially examined the time to all cause mortality from dialysis initiation for acute vs. chronic start patients and for CVC vs. PD/AVF access. After excluding acute start patients, the primary outcome of this study was to analyze the time to death for CVC vs. PD/AVF access. Patient survival was censored at transplantation or date of last follow-up (31 Mar 2011).

### Statistical analysis

Results were reported as counts and percentages for categorical variables, mean ± standard deviation for normally distributed continuous variables and median and interquartile range for non-normally distributed continuous variables. Categorical variables were compared using the Fisher’s Exact Test. Continuous variables were compared with t-tests and the Wilcoxon Rank Sum Test for normally and non-normally distributed variables, respectively. In addition, we performed an analysis of baseline characteristics restricted to chronic start patients, using the same statistical methods outlined above. Missing laboratory data was addressed using regression mean imputation. Complete variables chosen to impute missing data were age, gender, CCI and referral days. The time to mortality was graphed using the Kaplan-Meier product limit method and adjusted Cox survival curves. Adjusted hazard ratios and 95 % confidence intervals (CI) were estimated from multivariable Cox proportional hazards models. Variables selected for inclusion in the models were based on clinical judgment and known predictors derived from the literature. These variables included age, CCI, ESRD due to diabetes, gender, referral time, albumin, hemoglobin, phosphate and eGFR [19-26]. Interactions were examined for all variables used in the Cox survival analysis. Proportionality was examined with the test based on scaled Schoenfeld residuals. In addition to the primary analysis, we performed sensitivity analyses without censoring at transplantation, and using an eGFR cut-off of 30 ml/min/1.73 m^2^. All statistical analyses were performed using STATA IC, version 12 (StataCorp, College Station, TX). A two-sided P value < 0.05 was considered statistically significant. This study was approved by the Queen Elizabeth II Health Sciences Centre research ethics board, our institutional research ethics committee.

## Results

### All patients

**Figure 1:**
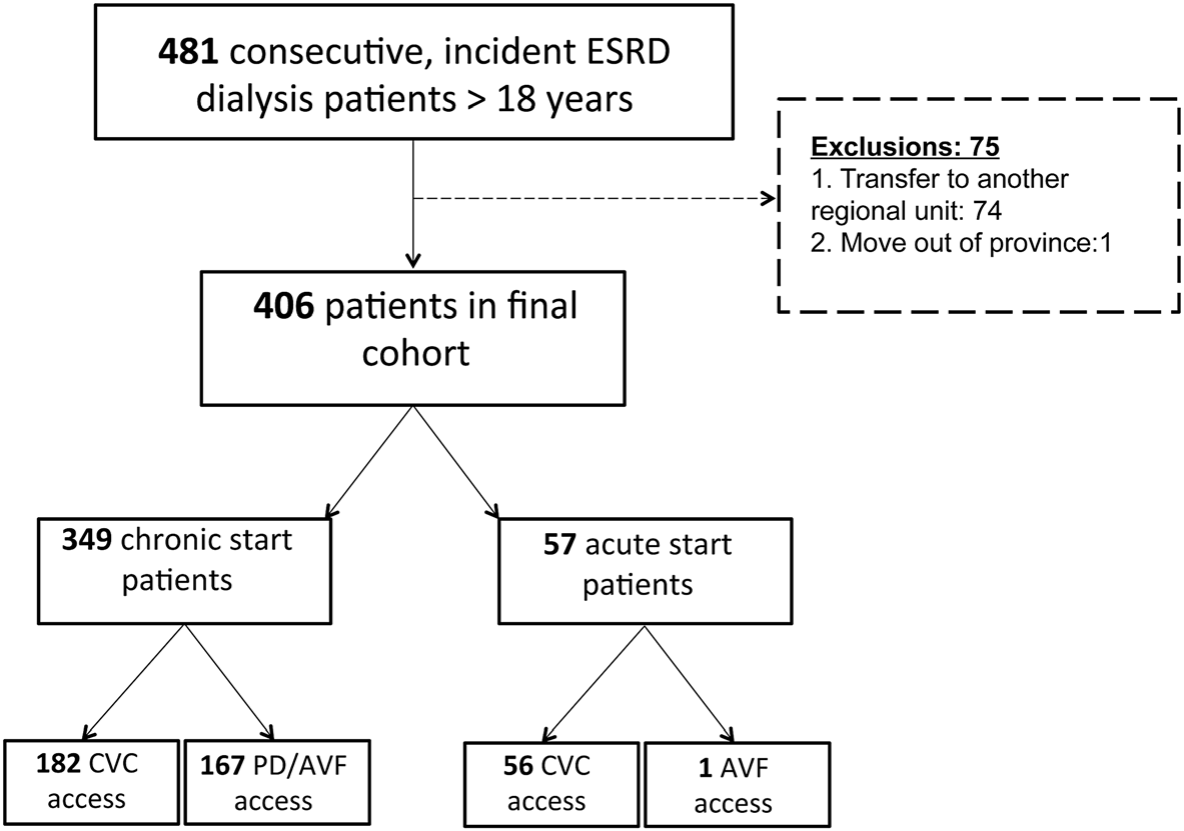
Cohort selection.

**Figure 2:**
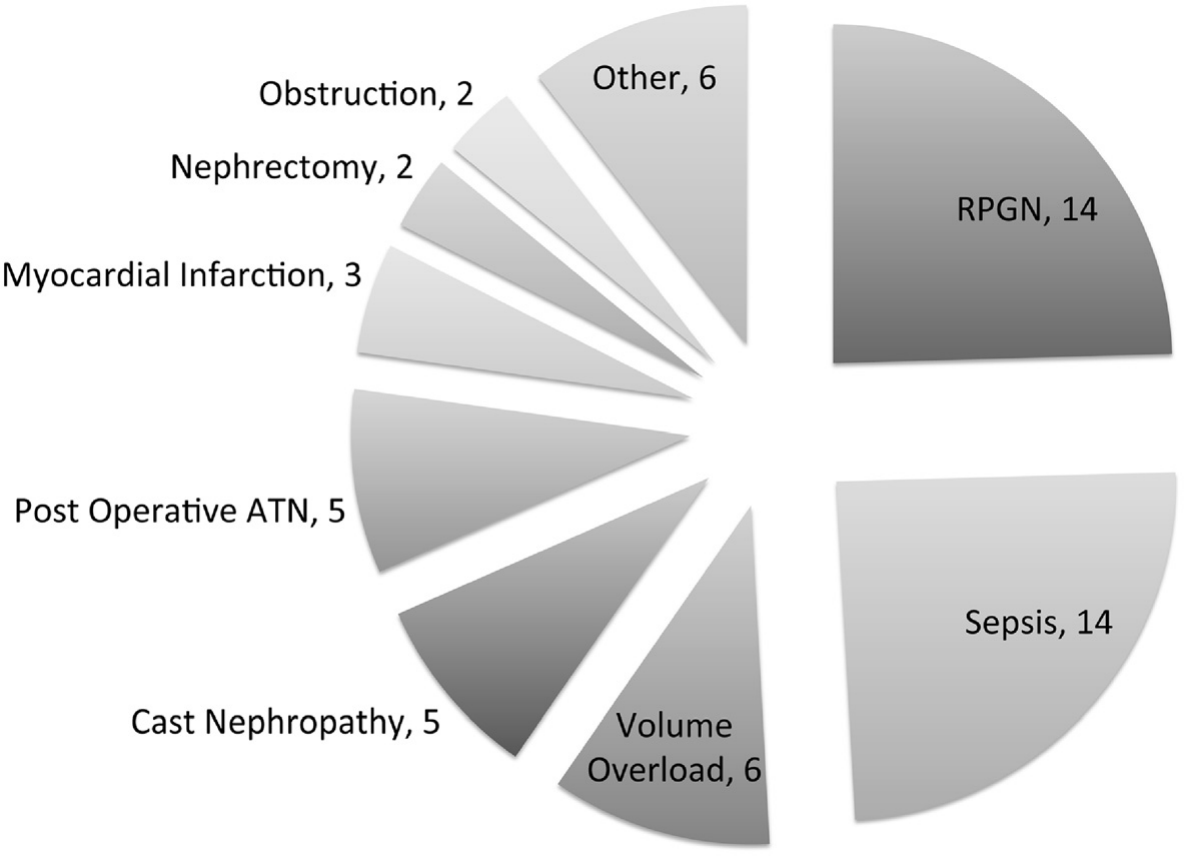
**Acute illness event leading to dialysis initiation.** Causes for category “other” were as follows: toxic acute tubular necrosis (ATN), atheroemboli, hypovolemic ischemic ATN, scleroderma renal crisis, tumour lysis syndrome (1 for each).

After exclusions (Figure 1), 406 patients were followed for a median of 22 months (IQR 13 to 35 months). 57 and 349 patients met the criteria for acute and chronic start, respectively. Amongst the chronic start patients, 335 fulfilled the definition based on eGFR criteria. One patient had an eGFR > 25 ml/min/1.73 m^2^ within three months of dialysis initiation, but without an acute illness event. The remaining 14 chronic start patients did not have eGFRs available between 12 and 3 months prior to starting dialysis, but small kidneys on ultrasound suggesting preexisting chronic kidney disease. Baseline characteristics of both groups are noted in Table 1. 36/57 and 9/57 acute start patients (63 % and 16 %) had eGFR measurements available at three and one month prior to dialysis initiation. The mean eGFR of these groups was 44 and 50 ml/min/1.73 m^2^, respectively. Of the remaining 12 patients without prior eGFR measurements, 8 had rapidly progressive glomerulonephritis (RPGN), 3 had multiple myeloma and 1 had pneumonia. The acute events leading to dialysis initiation in all acute start patients are noted in Figure 2.

**Table 1:** Baseline characteristics of acute and chronic start patients

	**Acute Start (n = 57)**	**Chronic Start (n = 349)**	**P**
Age at dialysis start, years, (mean ± SD)	71 ± 12.0	62 ± 16	<0.001
Male gender, n (%)	32 (57)	202 (58)	0.89
Caucasian race, n (%)	50 (88)	323 (93)	0.20
**Dialysis Access, n (%)**			
CVC	56 (98)	182 (52)	<0.001
AVF	1 (2)	95 (27)	<0.001
PD catheter	0	72 (21)	<0.001
**Cause of ESRD, n (%)**			
Diabetes	8 (14)	110 (32)	0.007
Polycystic kidney disease	2 (4)	30 (9)	0.29
Glomerulonephritis	10 (18)	44 (13)	0.53
Referral time before dialysis start, days, (median, IQR)	3, 1 to 33	1087, 443 to 2571	<0.001
Referral < 3 months, n (%)	47 (82)	34 (10)	<0.001
**Comorbidity**			
CCI (median, IQR)	5, 3 to 7	4, 2 to 6	0.03
Diabetes, n (%)	23 (41)	154 (44)	0.38
Coronary artery disease, n (%)	15 (27)	100 (29)	0.73
Congestive heart failure, n (%)	17 (30)	77 (22)	0.18
Peripheral vascular disease n (%)	14 (25)	78 (22)	0.73
Malignancy^a^, n (%)	17 (30)	34 (10)	<0.001
**Laboratory Values**			
Hemoglobin, g/L, (mean ± SD)	93 ± 15	100 ± 17	0.004
Albumin, g/L, (median, IQR)	26, 23 to 30	33, 28 to 36	<0.001
Phosphate, mmol/L, (median, IQR)	1.9, 1.4 to 2.6	1.8, 1.5 to 2.2	0.33
Imputed phosphate^b^	1.9, 1.5 to 2.6	1.9, 1.5 to 2.6	0.26
eGFR, ml/min/1.73 m^2^, (median, IQR)	6.8, 5.0 to 9.0	7.8, 6.0 to 10	0.01
Imputed eGFR^c^	6.8, 5.0 to 9.0	7.8, 6.0 to 10	0.01

a: Including hematologic or non-hematologic, excluding non-melanoma skin cancer.

b: 31 missing values imputed.

c: 3 missing values imputed.

**Figure 3:**
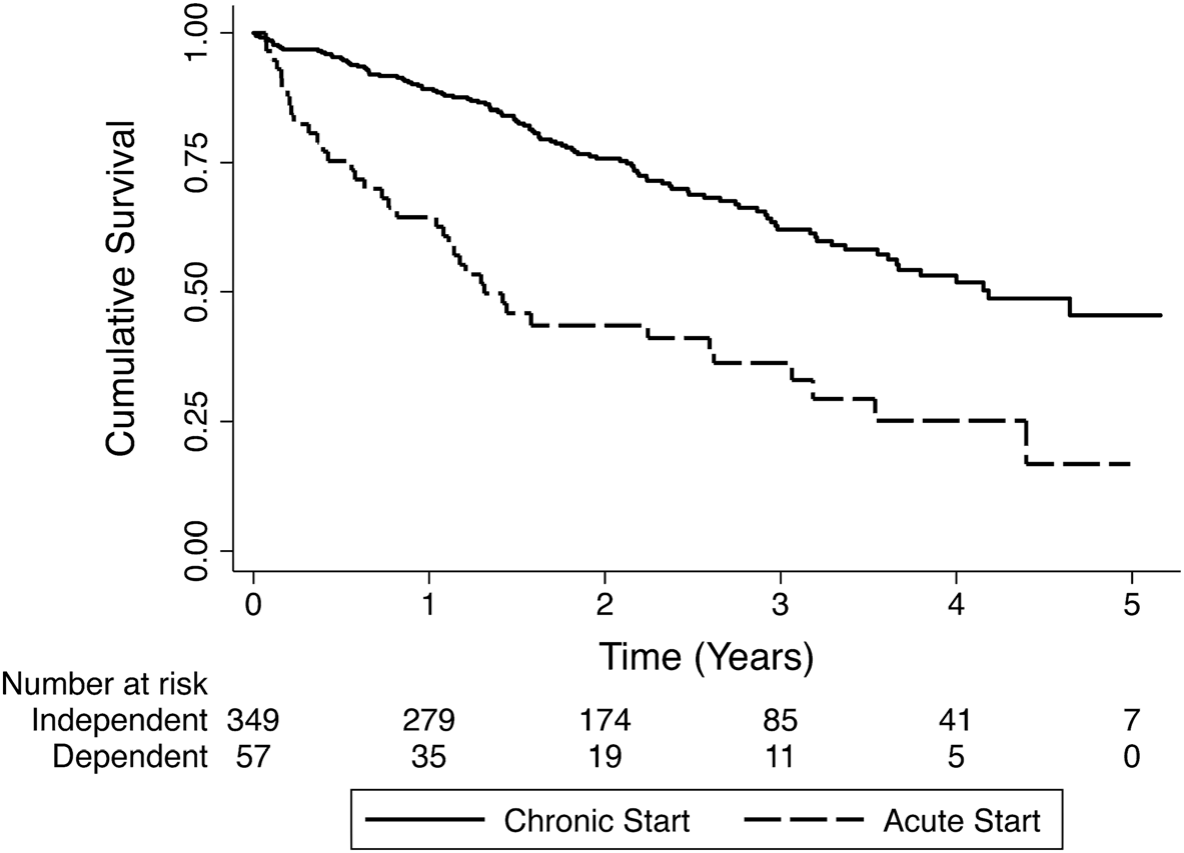
Kaplan Meier survival curves for acute and chronic start patients.

At last follow up, there were 62 kidney transplants and 148 deaths. 4/38 acute and 2/110 chronic start deaths were attributed to catheter-related sepsis (11 % and 2 % respectively). Causes of death are noted in Table 2. The two chronic start patients that died of catheter-related sepsis were 68 and 86 years of age. The first had a failed attempt at fistula creation prior to dialysis, and the latter chose PD but had an absolute contraindication due to a lack of home support and dementia. One additional chronic start patient (86 years of age), died from complications related to the CVC (superior vena cava thrombosis). This patient refused fistula creation prior to dialysis initiation. In the fully adjusted Cox model, an acute dialysis start was associated with shorter time to mortality compared to a chronic start (HR 1.84, 95 % CI [1.19-2.85], Table 3). Kaplan Meier survival curves for acute and chronic start patients are noted in Figure 3.

**Table 2:** Cause of death for acute and chronic start patients

**Cause**	**Acute Start (n = 38)**	**Chronic Start (n = 110)**
Catheter-related sepsis	4	2
Sepsis: other source	7	10
Cardiac (valvular, arrest, congestive heart failure, acute coronary syndrome)	7	30
Neurologic (stroke, seizure, delirium, demyelinating, dementia)	6	11
Non myeloma cancer		9
Multiple myeloma	3	1
Withdrawal due to quality of life	5	20
Gastrointestinal illness (bleed, obstruction)	3	3
Unknown	2	16
Respiratory failure		2
Other	1^a^	6^b^

a: Drug reaction.

b: Cirrhosis, failed bone marrow transplant, suicide, calciphylaxis, refractory hypotension, superior vena cava thrombosis.

**Table 3:** Cox survival analysis for acute vs. chronic start patients

**Model**	**HR [95 % CI]**	**P**
Unadjusted	2.83 [1.95 to 4.09]	<0.001
Model 1^a^	2.56 [1.76 to 3.71]	<0.001
Model 2^b^	2.35 [1.59 to 3.47]	<0.001
Model 3^c^	2.14 [1.42 to 3.22]	<0.001
Model 4^d^	1.84 [1.19 to 2.85]	0.006

a: Adjusted for age and gender.

b: Adjusted for factors in a. CCI and ESRD due to diabetes.

c: Adjusted for factors in b. and referral days.

d: Adjusted for factors in c. hemoglobin, albumin, phosphate and eGFR.

### CVC associated mortality

**Figure 4:**
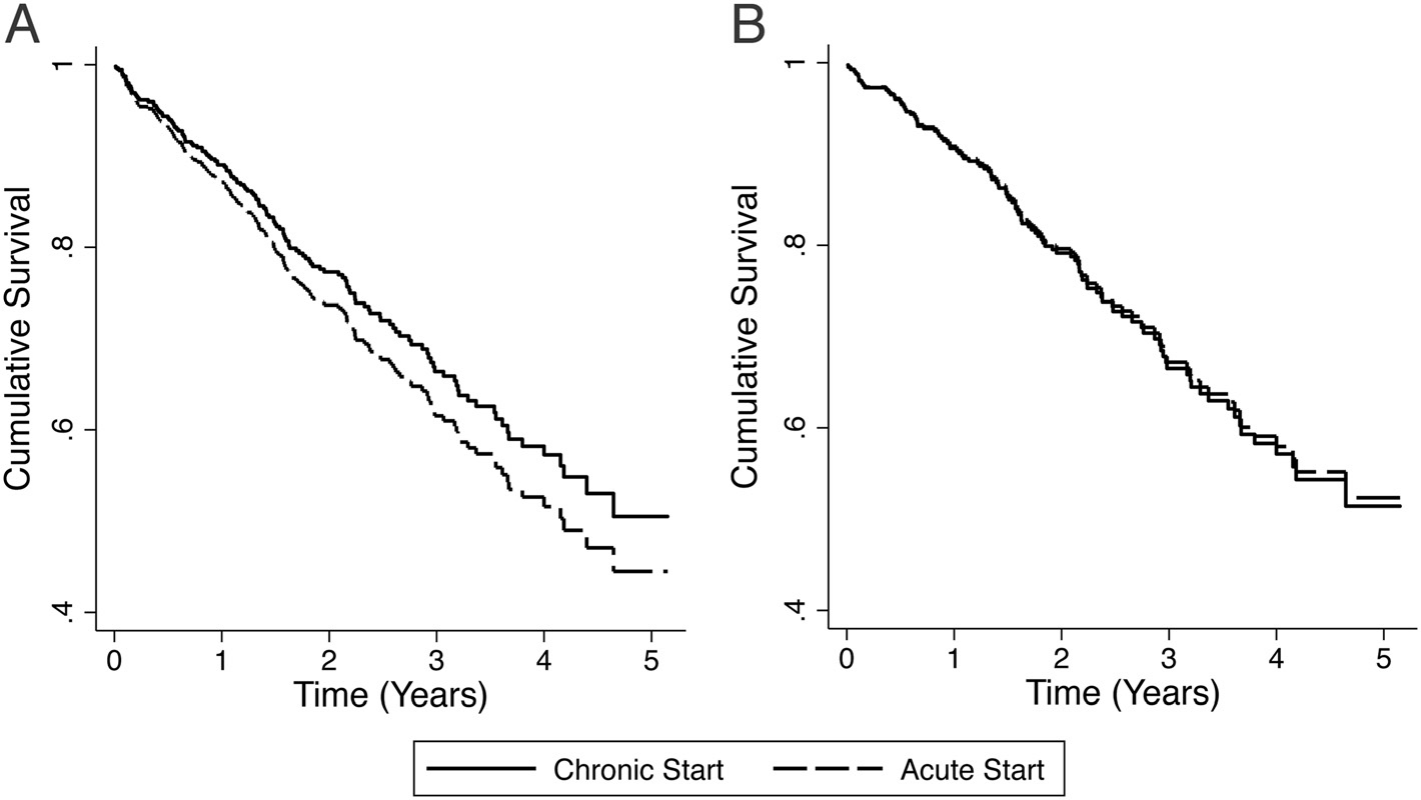
Adjusted Cox survival curves for CVC vs. PD catheter/AVF access in A) all patients and B) chronic start patients.

In the fully adjusted Cox model, the mortality HR for CVC vs. PD/AVF access was 1.19 (95 % CI, [0.80-1.77]). After excluding all acute starts, 349 chronic start patients remained for further analysis. Of these patients, 182 (52 %) initiated dialysis with a CVC. Baseline characteristics of patients dialyzing with a CVC vs. alternative access (PD catheter or AVF) are noted in Table 4. In the fully adjusted Cox model for chronic dialysis start patients, the mortality HR for CVCs vs. PD/AVF access was 1.03 (95 % CI [0.67 to 1.57], Table 5). Adjusted Cox survival curves for CVC vs. PD catheter/AVF access in the complete and restricted cohort are noted in Figure 4.

**Table 4:** Baseline characteristics of chronic start dialysis patients stratified by access

**Variable**	**CVC access (n = 182)**	**PD or fistula access (n = 167)**	**P**
Age at dialysis start, years, (mean ± SD)	62 ± 16.0	62 ± 16	0.95
Male gender, n (%)	96 (53)	106 (63)	0.05
Caucasian race, n (%)	166 (91)	157 (94)	0.42
**Dialysis Access, n (%)**			
CVC	56 (98)	182 (52)	<0.001
AVF	1 (2)	95 (27)	<0.001
PD catheter	0	72 (21)	<0.001
**Cause of ESRD, n (%)**			
Diabetes	64 (35)	46 (28)	0.14
Polycystic kidney disease	9 (5)	21 (13)	0.01
Glomerulonephritis	23 (13)	22 (13)	1.00
Referral time before dialysis start, days, (median, IQR)	734, 196 to 1966	1623, 687 to 3115	<0.001
Referral < 3 months, n (%)	32 (18)	2 (1)	<0.001
**Comorbidity**			
CCI (median, IQR)	5, 3 to 7	3, 2 to 6	<0.001
Diabetes, n (%)	91 (50)	63 (38)	0.02
Coronary artery disease, n (%)	62 (34)	38 (23)	0.02
Congestive heart failure, n (%)	44 (24)	33 (20)	0.37
Peripheral vascular disease n (%)	54 (30)	24 (14)	0.001
Malignancy^a^, n (%)	23 (13)	11 (7)	0.07
**Laboratory Values**			
Hemoglobin, g/L, (mean ± SD)	96 ± 17	105 ± 17	<0.001
Albumin, g/L, (median, IQR)	31, 25 to 34	34, 31 to 38	<0.001
Phosphate, mmol/L, (median, IQR)	1.8, 1.5 to 2.3	1.8, 1.5 to 2.2	0.19
Imputed phosphate^b^	1.9, 1.6 to 2.3	1.8, 1.5 to 2.1	0.13
eGFR, ml/min/1.73 m^2^, (median, IQR)	7.2, 5.8 to 9.7	8.1, 6.2 to 10.9	0.01
Imputed eGFR^c^	7.3, 5.8 to 9.6	8.1, 6.2 to 10.9	0.01

a: Including hematologic or non-hematologic, excluding non-melanoma skin CA.

b: 21 missing values imputed.

c: 3 missing values imputed.

**Table 5:** Cox survival analysis for CVC vs. AVF/PD catheter access

**Model**	**HR [95 % CI]**	**P**
**Complete cohort (n = 406)**		
Unadjusted	1.70 [1.20 to 2.41]	0.003
Model 1^a^	1.73 [1.22 to 2.45]	0.002
Model 2^b^	1.55 [1.08 to 2.22]	0.02
Model 3^c^	1.41 [0.97 to 2.05]	0.07
Model 4^d^	1.19 [0.80 to 1.77]	0.40
Model 4^d^ without censoring at transplantation	1.16 [0.78 to 1.73]	0.45
**Excluding acute start patients (n = 349)**		
Unadjusted	1.36 [0.93 to 2.00]	0.11
Model 1^a^	1.42 [0.97 to 2.09]	0.07
Model 2^b^	1.28 [0.87 to 1.90]	0.21
Model 3^c^	1.23 [0.83 to 1.84]	0.30
Model 4^d^	1.03 [0.67 to 1.57]	0.91
Model 4^d^ without censoring at transplantation	0.99 [0.64 to 1.51]	0.95
**Excluding acute start patients with an eGFR > 30 ml/min/1.73 m**^**2**^**(n = 359)**		
Unadjusted	1.42 [0.97 to 2.06]	0.07
Model 1^a^	1.46 [1.00 to 2.13]	0.05
Model 2^b^	1.32 [0.89 to 1.94]	0.16
Model 3^c^	1.26 [0.85 to 1.87]	0.24
Model 4^d^	1.05 [0.69 to 1.61]	0.81
Model 4^d^ without censoring at transplantation	1.02 [0.67 to 1.55]	0.94

a: Adjusted for age and gender.

b: Adjusted for factors in a. CCI and ESRD due to diabetes.

c: Adjusted for factors in b. and referral days.

d: Adjusted for factors in c. hemoglobin, albumin, phosphate and eGFR.

## Discussion

In this single center study of consecutive incident dialysis patients, we identified that a proportion of patients with a normal eGFR or early stage CKD (1 to early stage 4), initiated dialysis after an illness that induced a permanent, rapid loss of eGFR. Virtually all of these patients used a CVC as initial dialysis access. While this acute start patient group made up only a small percentage of all dialysis starts, they were at an increased risk for mortality compared to chronic start patients. Finally, CVCs were associated with a trend to increased mortality, but amongst chronic dialysis starts, the mortality risk of CVCs was attenuated and comparable to patients with alternative access.

For the chronic start patients, there are potential reasons that dialysis access was not associated with increased mortality, a finding in contrast to large registry studies [2,6]. An association between mortality and AKI/ACKD leading to ESRD has been demonstrated in previous studies [13,14,27]. The majority of these patients initiate dialysis with a CVC. However, even patients with non-dialysis requiring AKI have a higher mortality risk compared to those without AKI [28]. This emphasizes that the association between CVC access and mortality (in AKI patients who do not recover kidney function) may be the result of AKI itself. Alternatively AKI or ACKD may negatively impact on other factors that have been known to influence mortality in dialysis patients, such as residual renal function [29,30]. If these patients are not excluded from studies of incident dialysis and mortality, the mortality risk of CVCs may be overestimated. Another consideration is that the comparable survival for CVC vs. AVF/PD catheter access after exclusion of acute starts may be related to access conversion. While it was not the intention of this study, it has been shown that conversion from a CVC to an AVF after dialysis initiation is associated with increased survival [31].

The suggestion that the CVC associated mortality may be overestimated is further supported in our analysis of cause of death. Only 7/148 patient deaths (5 %) were clearly a direct result of the CVC itself (infection or vascular thrombosis). 4 of the 7 patient deaths were acute starts, a population who may be at an increased risk of hospital acquired bacteremia from other sources. While cause of death may have been subject to classification error, we were able to corroborate data with electronic records in this study. In contrast, registry studies may not always have access to individual records to confirm or refute cause of death [32].

However, even if CVC related mortality was underestimated in this study, complete avoidance of CVCs may be unrealistic and unachievable for a large number of incident dialysis patients. The three chronic start patients who died of catheter sepsis had largely unavoidable reasons for CVC use including previous access failure and patient refusal of alternative access. Furthermore, a recent study determined that the majority of late referred ESRD dialysis starts are unavoidable [33], and it can be assumed that many of these patients start dialysis with a CVC. This assumption was confirmed in our study, namely, that a sizeable proportion of patients develop ESRD after a rapid loss of eGFR, a setting in which alternative access is not anticipated. The notion that CVCs should be avoided at all costs also needs to be reconsidered in light of AVF success rates. Fistulas continue to have a high primary non-function rate [34-36]. The utility of AVFs is also questioned in many older patients. This is in part due to the high competing risk of death prior to dialysis access and the significant early mortality risk of elderly CKD patients with functioning access after the start of dialysis [37,38]. Overall, these scenarios suggest that while AVF and PD catheter access are preferred, there will still be considerable numbers of dialysis starts that will not be optimal despite our best efforts [39,40].

The findings of our study need to be compared to other cohort studies examining the association between dialysis access, acute start and mortality. The findings in our study were similar to a recent analysis of Veterans Affairs (VA) patients in which a “catastrophic loss of eGFR” (defined as loss from levels >60 ml/min/1.73 m^2^ within 6 months or less) was associated with early mortality [41]. While those patients initiated dialysis at a higher eGFR than our study (in part because the definition of predialysis eGFR was different), both studies highlight the importance of a rapid eGFR decline and its impact on mortality. In a large French REIN registry analysis of incident dialysis patients with congestive heart failure (CHF), despite excluding unplanned dialysis starts, CVC use was still associated with a statistically significant mortality HR of 1.35 (95 % CI 1.22-1.49) [42]. However, unlike the REIN study, our cohort had both CHF and non-CHF patients. In addition, the definition of an acute start differs from the unplanned definition used in REIN cohort studies [42,43]. We only classified AKI/ACKD patients who started dialysis as “acute starts”, but not those with previously undiagnosed or uninvestigated late stage CKD who presented late. While speculative, the latter may be a group of “survivors” (by virtue of being able to survive to the point of getting dialysis access) that would have been excluded from the REIN chronic cohort of planned dialysis starts.

There are limitations to this study. Three-month eGFR measurements were not available in all patients and ultrasound or patient history may be inaccurate in differentiating acute and chronic kidney disease. However, using the criteria in our definition, and considering the acute events in this patient group (primarily RPGN and myeloma) it was not illogical to classify patients as an acute start when eGFRs were not readily available. While the accuracy of our ESRD database is enhanced by its use of detailed electronic records, we acknowledge that there may have been some patients with AKI and early mortality that were misclassified as ESRD patients, or ESRD acute starts that were not included in this study. Because of a lack of frequent eGFR measurements in all patients, it was not possible to accurately capture the rate of eGFR decline and incorporate it into the definition. Some authorities may advocate for placement of a fistula or PD catheter at an eGFR >25 ml/min/1.73 m^2^ if a patient were experiencing a rapid decline in eGFR. However, a sensitivity analysis using a more conservative eGFR cutoff of >30 ml/min/1.73 m^2^ (corresponding to the lower limit of stage 3 CKD), did not change the results of our study. A final limitation of our definition of acute start is that fistulas or PD catheters can be placed and accessed within 3 months of identifying a need for dialysis. However, our population also required an acute illness event to precipitate the decline in eGFR prior to dialysis initiation. Therefore, most physicians would be unprepared to institute optimal access placement in this population. This would be true for patients with a recent cardiac/septic event or admission in an individual with potentially reversible AKI (i.e. myeloma or RPGN). In addition to the study population, there are other limitations inherent to the study design. Despite controlling for multiple variables, there is the possibility of residual confounding and bias. It is possible that a larger study may detect a small, persistent, residual early mortality risk associated with CVCs. Furthermore, while we attempted to capture all of the CVC related deaths, 15 % of deaths amongst chronic start patients were of unknown cause. It is possible that additional catheter-related septic deaths were missed in this group.

It should be emphasized that while this study suggests that the mortality attributed to CVCs needs to be examined after consideration of an “acute start”, we are not suggesting that CVCs are optimal dialysis access. We acknowledge that our patients may have developed CVC complications that indirectly led to alternative causes of death or morbidity. Moreover, given CVCs are associated with significant morbidity and cost [44-47], there should be concerted efforts to obtain alternative access in prevalent dialysis patients.

## Conclusion

In summary, an acute dialysis start is associated with increased mortality and exclusion of this population attenuates the mortality risk of CVC use. Prior registry reports may have overestimated CVC mortality, as acute starts were not considered. Additional studies should be conducted to further establish the potential modifying effect of an acute start on CVC associated mortality.

## Abbreviations

ACKD: Acute on Chronic Kidney Disease; AKI: Acute Kidney Injury; AVF: Arteriovenous Fistula; CCI: Charlson Comorbidity Index; CKD: Chronic Kidney Disease; CORR: Canadian Organ Replacement Register; CSN: Canadian Society of Nephrology; CVC: Central Venous Catheter; ESRD: End Stage Renal Disease; eGFR: Estimated Glomerular Filtration Rate; HD: Hemodialysis; HR: Hazard Ratio; KDOQI: Kidney Disease Outcomes Quality Initiative; PD: Peritoneal Dialysis; RPGN: Rapidly Progressive Glomerulonephritis.

## Competing interests

The authors declare that they have no competing interests.

## Authors’ contributions

Karthik Tennankore contributed to the study design, data collection, data analysis and interpretation of the results. He also drafted the initial manuscript.

Steven Soroka contributed to data acquisition and content revision for the final manuscript.

Bryce Kiberd devised the study and contributed to data collection and analysis. He was also involved in content revision for the final manuscript.

All three authors provided final approval of the version to be published.

## Pre-publication history

The pre-publication history for this paper can be accessed here:

http://www.biomedcentral.com/1471-2369/13/72/prepub

## References

[B1] Astor BC Eustace JA Powe NR Klag MJ Fink NE Coresh J Type of vascular access and survival among incident hemodialysis patients: the Choices for Healthy Outcomes in Caring for ESRD (CHOICE) Study J Am Soc Nephrol 2005 16 5 1449 1455 10.1681/ASN.2004090748 15788468

[B2] Moist LM Trpeski L Na Y Lok CE Increased hemodialysis catheter use in Canada and associated mortality risk: data from the Canadian Organ Replacement Registry 2001-2004 Clin J Am Soc Nephrol 2008 3 6 1726 1732 10.2215/CJN.01240308 18922993 PMC2572294

[B3] Pisoni RL Arrington CJ Albert JM Ethier J Kimata N Krishnan M Rayner HC Saito A Sands JJ Saran R Facility hemodialysis vascular access use and mortality in countries participating in DOPPS: an instrumental variable analysis Am J Kidney Dis 2009 53 3 475 491 10.1053/j.ajkd.2008.10.043 19150158

[B4] Polkinghorne KR McDonald SP Atkins RC Kerr PG Vascular access and all-cause mortality: a propensity score analysis J Am Soc Nephrol 2004 15 2 477 486 10.1097/01.ASN.0000109668.05157.05 14747396

[B5] Astor BC Eustace JA Powe NR Klag MJ Sadler JH Fink NE Coresh J Timing of nephrologist referral and arteriovenous access use: the CHOICE Study Am J Kidney Dis 2001 38 3 494 501 10.1053/ajkd.2001.26833 11532680

[B6] Perl J Wald R McFarlane P Bargman JM Vonesh E Na Y Jassal SV Moist L Hemodialysis vascular access modifies the association between dialysis modality and survival J Am Soc Nephrol 2011 22 6 1113 1121 10.1681/ASN.2010111155 21511830 PMC3103730

[B7] Jindal K Chan CT Deziel C Hirsch D Soroka SD Tonelli M Culleton BF Hemodialysis clinical practice guidelines for the Canadian Society of Nephrology J Am Soc Nephrol 2006 17 3 Suppl 1 1 27 10.1681/ASN.2005121372 16497879

[B8] 2006 Updates Clinical Practice Guidelines and Recommendations: Hemodialysis Adequacy, Peritoneal Dialysis Adequacy, Vascular Accesshttp://www.kidney.org/professionals/kdoqi/guidelines

[B9] Sidawy AN Spergel LM Besarab A Allon M Jennings WC Padberg FT Murad MH Montori VM O'Hare AM Calligaro KD The Society for Vascular Surgery: clinical practice guidelines for the surgical placement and maintenance of arteriovenous hemodialysis access J Vasc Surg 2008 48 5 2 25 10.1016/j.jvs.2008.08.042 19000589

[B10] Figueiredo A Goh BL Jenkins S Johnson DW Mactier R Ramalakshmi S Shrestha B Struijk D Wilkie M Clinical practice guidelines for peritoneal access Perit Dial Int 2010 30 4 424 429 10.3747/pdi.2010.00087 20628103

[B11] Ponce D Balbi AL Peritoneal dialysis in acute kidney injury: a viable alternative Perit Dial Int 2011 31 4 387 389 10.3747/pdi.2011.00312 21799052

[B12] Povlsen JV Ivarsen P How to start the late referred ESRD patient urgently on chronic APD Nephrol Dial Transplant 2006 21 Suppl 2 56 59 10.1093/ndt/gfl192 16825263

[B13] Bhandari S Turney JH Survivors of acute renal failure who do not recover renal function QJM 1996 89 6 415 421 10.1093/qjmed/89.6.415 8758044

[B14] Hsu CY Chertow GM McCulloch CE Fan D Ordonez JD Go AS Nonrecovery of kidney function and death after acute on chronic renal failure Clin J Am Soc Nephrol 2009 4 5 891 898 10.2215/CJN.05571008 19406959 PMC2676192

[B15] Descamps C Labeeuw M Trolliet P Cahen R Ecochard R Pouteil-Noble C Villar E Confounding factors for early death in incident end-stage renal disease patients: Role of emergency dialysis start Hemodial Int 2011 15 1 23 29 10.1111/j.1542-4758.2010.00513.x 21223483

[B16] Lee P Johansen K Hsu CY End-stage renal disease preceded by rapid declines in kidney function: a case series BMC Nephrol 2011 12 5 10.1186/1471-2369-12-5 21284877 PMC3042936

[B17] Emamian SA Nielsen MB Pedersen JF Ytte L Kidney dimensions at sonography: correlation with age, sex, and habitus in 665 adult volunteers AJR American journal of roentgenology 1993 160 1 83 86 8416654 10.2214/ajr.160.1.8416654

[B18] Charlson ME Pompei P Ales KL MacKenzie CR A new method of classifying prognostic comorbidity in longitudinal studies: development and validation J Chronic Dis 1987 40 5 373 383 10.1016/0021-9681(87)90171-8 3558716

[B19] Bradbury BD Fissell RB Albert JM Anthony MS Critchlow CW Pisoni RL Port FK Gillespie BW Predictors of early mortality among incident US hemodialysis patients in the Dialysis Outcomes and Practice Patterns Study (DOPPS) Clin J Am Soc Nephrol 2007 2 1 89 99 17699392 10.2215/CJN.01170905

[B20] Nitsch D Burden R Steenkamp R Ansell D Byrne C Caskey F Roderick P Feest T Patients with diabetic nephropathy on renal replacement therapy in England and Wales QJM 2007 100 9 551 560 10.1093/qjmed/hcm062 17681992

[B21] Noordzij M Korevaar JC Dekker FW Boeschoten EW Bos WJ Krediet RT Bossuyt PM Geskus RB Mineral metabolism and mortality in dialysis patients: a reassessment of the K/DOQI guideline Blood Purif 2008 26 3 231 237 10.1159/000118847 18305386

[B22] Plantinga LC Fink NE Levin NW Jaar BG Coresh J Levey AS Klag MJ Powe NR Early, intermediate, and long-term risk factors for mortality in incident dialysis patients: the Choices for Healthy Outcomes in Caring for ESRD (CHOICE) Study Am J Kidney Dis 2007 49 6 831 840 10.1053/j.ajkd.2007.03.017 17533026

[B23] Wright S Klausner D Baird B Williams ME Steinman T Tang H Ragasa R Goldfarb-Rumyantzev AS Timing of dialysis initiation and survival in ESRD Clin J Am Soc Nephrol 2010 5 10 1828 1835 10.2215/CJN.06230909 20634325 PMC2974384

[B24] Foley RN Parfrey PS Harnett JD Kent GM Murray DC Barre PE The impact of anemia on cardiomyopathy, morbidity, and and mortality in end-stage renal disease Am J Kidney Dis 1996 28 1 53 61 10.1016/S0272-6386(96)90130-4 8712222

[B25] Smart NA Titus TT Outcomes of early versus late nephrology referral in chronic kidney disease: a systematic review Am J Med 2011 124 11 1073 1080 10.1016/j.amjmed.2011.04.026 22017785

[B26] Hemmelgarn BR Manns BJ Quan H Ghali WA Adapting the Charlson Comorbidity Index for use in patients with ESRD Am J Kidney Dis 2003 42 1 125 132 10.1016/S0272-6386(03)00415-3 12830464

[B27] Wu VC Huang TM Lai CF Shiao CC Lin YF Chu TS Wu PC Chao CT Wang JY Kao TW Acute-on-chronic kidney injury at hospital discharge is associated with long-term dialysis and mortality Kidney Int 2011 80 11 1222 1230 10.1038/ki.2011.259 21832983

[B28] Lafrance JP Miller DR Acute kidney injury associates with increased long-term mortality J Am Soc Nephrol 2010 21 2 345 352 10.1681/ASN.2009060636 20019168 PMC2834549

[B29] Shafi T Jaar BG Plantinga LC Fink NE Sadler JH Parekh RS Powe NR Coresh J Association of residual urine output with mortality, quality of life, and inflammation in incident hemodialysis patients: the Choices for Healthy Outcomes in Caring for End-Stage Renal Disease (CHOICE) Study Am J Kidney Dis 2010 56 2 348 358 10.1053/j.ajkd.2010.03.020 20605303 PMC2910835

[B30] Bargman JM Thorpe KE Churchill DN Relative contribution of residual renal function and peritoneal clearance to adequacy of dialysis: a reanalysis of the CANUSA study J Am Soc Nephrol 2001 12 10 2158 2162 11562415 10.1681/ASN.V12102158

[B31] Lacson E Wang W Lazarus JM Hakim RM Change in vascular access and mortality in maintenance hemodialysis patients Am J Kidney Dis 2009 54 5 912 921 10.1053/j.ajkd.2009.07.008 19748717

[B32] Chan KE Maddux FW Tolkoff-Rubin N Karumanchi SA Thadhani R Hakim RM Early outcomes among those initiating chronic dialysis in the United States Clin J Am Soc Nephrol 2011 6 11 2642 2649 10.2215/CJN.03680411 21959599 PMC3359565

[B33] Udayaraj UP Haynes R Winearls CG Late presentation of patients with end-stage renal disease for renal replacement therapy–is it always avoidable? Nephrol Dial Transplant 2011 26 11 3646 3651 10.1093/ndt/gfr164 21454353

[B34] Schinstock CA Albright RC Williams AW Dillon JJ Bergstralh EJ Jenson BM McCarthy JT Nath KA Outcomes of arteriovenous fistula creation after the fistula first initiative Clin J Am Soc Nephrol 2011 6 8 1996 2002 10.2215/CJN.11251210 21737851 PMC3156429

[B35] Biuckians A Scott EC Meier GH Panneton JM Glickman MH The natural history of autologous fistulas as first-time dialysis access in the KDOQI era J Vasc Surg 2008 47 2 415 421 10.1016/j.jvs.2007.10.041 18241764

[B36] Lazarides MK Georgiadis GS Antoniou GA Staramos DN A meta-analysis of dialysis access outcome in elderly patients J Vasc Surg 2007 45 2 420 426 10.1016/j.jvs.2006.10.035 17264030

[B37] Vachharajani TJ Moossavi S Jordan JR Vachharajani V Freedman BI Burkart JM Re-evaluating the Fistula First Initiative in Octogenarians on Hemodialysis Clin J Am Soc Nephrol 2011 6 7 1663 1667 10.2215/CJN.05830710 21685023

[B38] Richardson AI Leake A Schmieder GC Biuckians A Stokes GK Panneton JM Glickman MH Should fistulas really be first in the elderly patient? J Vasc Access 2009 10 3 199 202 19670174 10.1177/112972980901000311

[B39] Mendelssohn DC Curtis B Yeates K Langlois S MacRae JM Semeniuk LM Camacho F McFarlane P Suboptimal initiation of dialysis with and without early referral to a nephrologist Nephrol Dial Transplant 2011 26 9 2959 2965 10.1093/ndt/gfq843 21282303

[B40] Mendelssohn DC Malmberg C Hamandi B An integrated review of "unplanned" dialysis initiation: reframing the terminology to "suboptimal" initiation BMC Nephrol 2009 10 22 10.1186/1471-2369-10-22 19674452 PMC2735745

[B41] O'Hare AM Batten A Burrows NR Pavkov ME Taylor L Gupta I Todd-Stenberg J Maynard C Rodriguez RA Murtagh FE Trajectories of kidney function decline in the 2 years before initiation of long-term dialysis Am J Kidney Dis 2012 59 4 513 522 10.1053/j.ajkd.2011.11.044 22305760 PMC3312937

[B42] Sens F Schott-Pethelaz AM Labeeuw M Colin C Villar E Survival advantage of hemodialysis relative to peritoneal dialysis in patients with end-stage renal disease and congestive heart failure Kidney Int 2011 80 9 970 977 10.1038/ki.2011.233 21775972

[B43] Couchoud C Moranne O Frimat L Labeeuw M Allot V Stengel B Associations between comorbidities, treatment choice and outcome in the elderly with end-stage renal disease Nephrol Dial Transplant 2007 22 11 3246 3254 10.1093/ndt/gfm400 17616533

[B44] Hoen B Paul-Dauphin A Hestin D Kessler M EPIBACDIAL: a multicenter prospective study of risk factors for bacteremia in chronic hemodialysis patients J Am Soc Nephrol 1998 9 5 869 876 9596085 10.1681/ASN.V95869

[B45] Manns B Tonelli M Yilmaz S Lee H Laupland K Klarenbach S Radkevich V Murphy B Establishment and maintenance of vascular access in incident hemodialysis patients: a prospective cost analysis J Am Soc Nephrol 2005 16 1 201 209 15563567 10.1681/ASN.2004050355

[B46] Wasse H Kutner N Zhang R Huang Y Association of initial hemodialysis vascular access with patient-reported health status and quality of life Clin J Am Soc Nephrol 2007 2 4 708 714 10.2215/CJN.00170107 17699486 PMC2728772

[B47] Ng LJ Chen F Pisoni RL Krishnan M Mapes D Keen M Bradbury BD Hospitalization risks related to vascular access type among incident US hemodialysis patients Nephrol Dial Transplant 2011 26 11 3659 3666 10.1093/ndt/gfr063 21372255

